# The Selection of Reliable Reference Genes for RT-qPCR Analysis of *Anisakis simplex* Sensu Stricto Gene Expression from Different Developmental Stages

**DOI:** 10.2478/s11686-020-00220-3

**Published:** 2020-06-02

**Authors:** Elżbieta Łopieńska-Biernat, Robert Stryiński, Łukasz Paukszto, Jan Paweł Jastrzębski, Karol Makowczenko

**Affiliations:** 1 Department of Biochemistry, Faculty of Biology and Biotechnology, University of Warmia and Mazury in Olsztyn, Oczapowskiego 1A, 10-719 Olsztyn, Poland; 2 Department of Plant Physiology, Genetics and Biotechnology, Faculty of Biology and Biotechnology, University of Warmia and Mazury in Olsztyn, Oczapowskiego 1A, 10-719 Olsztyn, Poland

**Keywords:** *Anisakis simplex*, RT-qPCR, Gene expression, Housekeeping gene

## Abstract

**Background:**

*Anisakis simplex* s. s. is a parasitic nematode with a complex life cycle in which humans can become accidental hosts by consuming raw or not fully cooked fish containing L3 larvae. The growing popularity of raw fish dishes has contributed to an increase in the incidence of anisakiasis, which has spurred scientific efforts to develop new methods for diagnosing and treating the disease and also to investigate the gene expression at different developmental stages of this parasite. The identification of reference genes suitable for the normalization of RT-qPCR data has not been studied with respect to *A. simplex* s. s.

**Methods:**

In the present study, eight candidate reference genes were analyzed in *A. simplex* s. s. at two different developmental stages: L3 and L4. The expression stability of these genes was assessed by geNorm and NormFinder softwares.

**Results:**

In general, our results identified translation elongation factor 1*α* (*ef-1α*) and peptidyl-prolyl isomerase 12 (*ppi12)* as the most stable genes in L3 and L4 developmental stages of *A. simplex* s. s. Validation of the selected reference genes was performed by profiling the expression of the nuclear hormone receptor gene (*nhr 48*) in different developmental stages.

**Conclusions:**

This first analysis selecting suitable reference genes for RT-qPCR in *A. simplex* s. s. will facilitate future functional analyses and deep mining of genetic resources in this parasitic nematode.

**Electronic Supplementary Material:**

The online version of this article (10.2478/s11686-020-00220-3) contains supplementary material, which is available to authorized users.

## Background

*Anisakis simplex* s. s. is a ubiquitous parasitic nematode. Adult parasites live in the stomach of fish-eating marine mammals, such as orcas, dolphins, seals and porpoises [1, 2]. *A. simplex* s. s. has a complex life cycle in which humans can become accidental hosts by consuming raw or not fully cooked fish containing L3 larvae [3]. Parasitic larvae produce proteolytic enzymes [4], penetrate gastrointestinal mucosa and cause mucosal inflammation known as anisakiasis [5]. Infections caused by *A. simplex* s. s. produce gastrointestinal symptoms and are often accompanied by mild allergic reactions. However, sudden and severe allergic reactions have been noted without gastric symptoms [6]. In several documented cases, allergic reactions to L3 larvae of *A. simplex* have been reported in humans after the consumption of fish processed at high or low temperature [7].

*A. simplex* as a fish-borne parasite responsible for human anisakiasis and allergic reactions around the world became an organism of scientific interest. The investigation of the expression of genes at different developmental stages of this parasite seems to be important. It will affect the better understanding of the molecular biology of these parasitic nematodes and will allow finding ways to overcome the disease caused by them. A real-time polymerase chain reaction (real-time PCR) is a valuable method to quantify gene expression [8] at different developmental stages [9, 10]. DNA quantification, in relative quantification PCR method, is based on the determination of differences in expression between the target and reference genes. The reason for using one or more reference genes is to reduce the influence of a number of variables during the real-time PCR reaction, such as RNA integrity and quantity, efficiency in cDNA synthesis, and PCR amplification [11]. However, the most important aspect of the real-time PCR experiment planning is the selection of the reference gene, whose expression must be stable for that specific organism we work with [12].

In this study, we aimed to evaluate the expression stability of the candidate reference genes in *A. simplex* s. s. at key developmental stages (L3 and L4) in anisakiasis etiology, using two different statistical algorithms. Additionally, the proposed reference genes were used to normalize the expression of a target gene in *A. simplex* s. s. to validate the obtained results.

## Materials and Methods

The experiment was performed on two developmental stages of *A. simplex* s. s.: L3 and L4. The L3 larvae were isolated from dead Baltic herrings (*Clupea harengus membras*) purchased at the market in Olsztyn and rinsed three times in sterile saline solution (0.9% NaCl).

All larvae (65) were assessed under stereomicroscope as belonging to the *A. simplex* species type I. Five of all larvae used in the experiment were subjected to taxonomic identification based on highly specific amplification of the genomic DNA fragment from the ITS1 / ITS2 region and isolated with the use of Xpure TM Cell & Tissue micro (A & A Biotechnology, Poland). Taxonomic identification was done with the use of Anis Sensitive Sniper Real-Time PCR kit according to the manufacturer’s protocol (A & A Biotechnology, Poland) as described before by Łopieńska-Biernat et al. [13]. The kit contains ready-to-use PCR reaction mixtures containing species-specific primers. To determine the species of the parasite, with isolated DNA, seven PCR reactions were performed in parallel (with each of the specific mixtures). The spreadsheet provided by the manufacturer after filling the Ct values obtained during real-time PCR shows which of the species’ DNA was in the tested sample.

Sixty larvae were divided into two equal groups of 30 specimens each. Thirty L3 larvae were cultured in vitro until they reached the L4 stage (6 days), according to the method described by Iglesias et al. [14]. The culture medium was replaced every 2 days. The experiment was performed in triplicate. Cultured L4 larvae and L3 isolated directly from the host were stabilized in the StayRNA buffer (A&A Biotechnology, Poland) and stored at − 80 °C for further analysis.

The *A. simplex* s. s. genome (ERS2790326) was used to determine the eight gene sequences of *A. simplex* potential reference genes (*gapdh*, *pdi*, *ppi12*, *β-tubulin*, *actin*, *ubiquitin*, *ef-1α*, *nhr 48*). The analysis was based on the reference gene sequences of *Caenorhabditis elegans, Ascaris suum*, *Toxocara canis*, *Loa loa* and *Brugia malayi* from GenBank and wormBase.org, as well as on the phylogenetic similarity using the MrBayes 3.2.2 application in Geneious v.3.8 [15]. These data were submitted to the public database (https://goo.gl/uPgcDn) and the sequences of genes determined for *A. simplex* s. s. were deposited in GenBank: *gapdh* (KM496565), *pdi* (KM496569), *ppi 12* (KM496568), *β-tubulin* (KP326559), *actin* (KP200883), *ubiquitin* (KM496564), *ef-1α* (KP326558) and *nhr48* (KR092170).

The total RNA of L3 and L4 was isolated with the Total RNA Mini Plus kit (A&A Biotechnology, Poland) according to the manufacturer’s instructions. cDNA was synthesized with 2 µg of RNA, oligo (dT) primers and reverse transcriptase from the TransScriba Kit (A&A Biotechnology, Poland). The primers used in the experiment were designed in the Primer3 (Table 1) (https://frodo.wi.mit.edu).

**Table 1:** The genes of *Anisakis simplex* s. s. and primers sequences used for qRT-PCR assay

Gene	NCBI No	Length (bp)	Sense primer	Antysense primer
actin	KP200883	168	TGGAGTGGTGCTTGACTCAG	TCACGAACAATCTCACGCTC
18s rRNA	U81575	177	TCACCAATCTCGGCTGACAA	GCACCAATAATGCGATTGTG
pdi	KM496569	151	ATGCCATTCGGAATCACTTC	CCACCTGGATCCAAGCTTTA
ppi 12	KM496568	218	GGCACGATATTCCACAGGAT	CTCCATAGATCGATGCACCA
ef-1α	KP326558	189	CACCGACTTCACCTCAGAGT	ACCACTCAGACTGCCTCTTC
ubiquitin	KM496564	151	TGAAAGAAGACGCAAACGTG	CGGATCAAAATGACCCACTT
β-tubulin	KP326559	280	GCTGCCACTGTCAGATAACG	ACATGGTTCCATTCCCTCGT
gapdh	KM496565	156	AGTCCACTGGTGTGTTCACG	CCTCATTGACTCCCATCACA
nhr48	KR092170	203	TCATTGCTTCGATCAGTGCG	GAAGCTGTTGACGCCCATAG

The real-time PCR was carried out with the use of the ABI PRISM 7500 thermocycler (Applied Biosystems, Poland) and the RT HS-PCR Mix SYBR B (A&A Biotechnology, Poland) according to the manufacturer’s instruction. The following program parameters were used for all amplifications: 95 °C for 10 min, followed by 40 three-step cycles: 95 °C for 15 s, 60 °C for 60 s, and 72 °C for 30 s. At the end of each program, the specificity of the primer sets was confirmed by melting curve analysis. Every gene was analyzed in triplicate during three experiments. Standard curves for each gene (copy number) in serial dilutions of 10^−1^, 10^−2^, 10^−3^, 10^−4^, 10^−5^ were plotted to evaluate the effectiveness of reaction amplification. The Ct values for each examined gene were means of three replicates.

The selection of the most stable reference genes for real-time PCR is based on the statistical softwares. In this study, to assess the stability of expression profiles of the proposed reference genes, we used two of the most popular ones: geNORM and NormFinder. The geNORM software, for every reference gene, determines the pairwise variation with all other proposed reference genes as the standard deviation of the logarithmically transformed expression values and defines the internal control gene-stability value—*M*. The lowest *M* value describes the gene with the most stable expression [16]. The NormFinder automatically calculates the stability value (SV) for all candidate reference genes tested among samples in the given groups. The software selects genes with the lowest SV to be considered as genes with the highest expression stability [17].

To confirm the stability of expression of the selected reference genes, verification experiment was carried out in samples from different developmental stages. Relative quantification of the nuclear hormone receptor family member *nhr-48* target gene was calculated using the 2^−ΔΔCt^ method [18]. The results were analyzed statistically in the Statistica 12.0 with the use of ANOVA and Tukey’s test at a significance level of *p* ≤ 0.05.

## Results and Discussion

The taxonomic identification, except microscopical overview, was based on highly specific amplification of the genomic DNA fragment of the ITS1/ITS2 region. The analysis showed that the larvae belong to the species *A. simplex* s. s. (Supplemental Table 1).

**Fig. 1:**
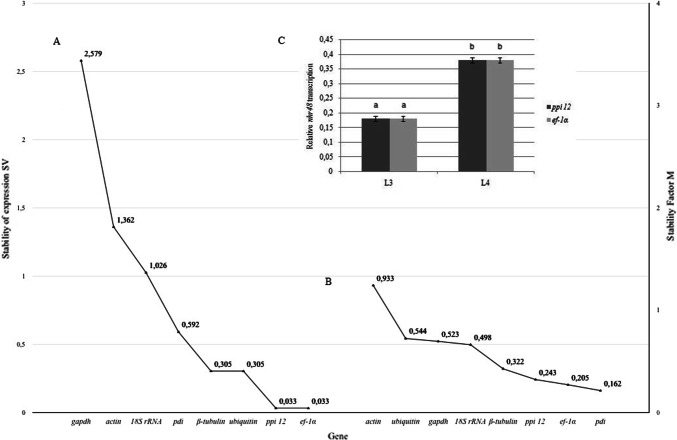
The average values of gene expression stability in *Anisakis simplex* s. s.*,* generated in NormFinder (**a**) and geNorm v.3.4 (**b**) and relative transcription level of *nhr-48* target gene normalized to expression profile of reference gene *ef-1α* and *ppi 12* (**c**). Explanation: mean ± SD. The different lowercase letters above the bar indicate significant differences (*p* ≤ 0.05)

The real-time PCR method used for quantifying gene expression levels requires suitable reference genes as internal controls. The present study is the first evaluating and validating a series of candidate reference genes which might be suitable for real-time PCR gene expression analysis in different developmental stages (L3 and L4) of *A. simplex* s. s. Gene candidates’ stability was assessed and validated by geNORM and NormFinder, where the relative values of quantitative data (2^(−ΔCт)^) were used to calculate, respectively, M and SV coefficients describing the stability of gene expression. The analysis with the NormFinder software showed that *pdi* (SV = 0.162) and *ef-1α* (SV = 0.205) were the most stable genes, whereas *actin* (SV = 0.933) was described as a gene with the least stable expression (Fig. 1a). The analysis in geNORM revealed that *ppi 12* (*M* = 0.033) and *ef-1α* (*M* = 0.033) were the most stable reference genes, whereas *gapdh* (*M* = 2.579) was the least stable reference gene for *A. simplex* s. s. (Fig. 1b).

**Fig. 2:**
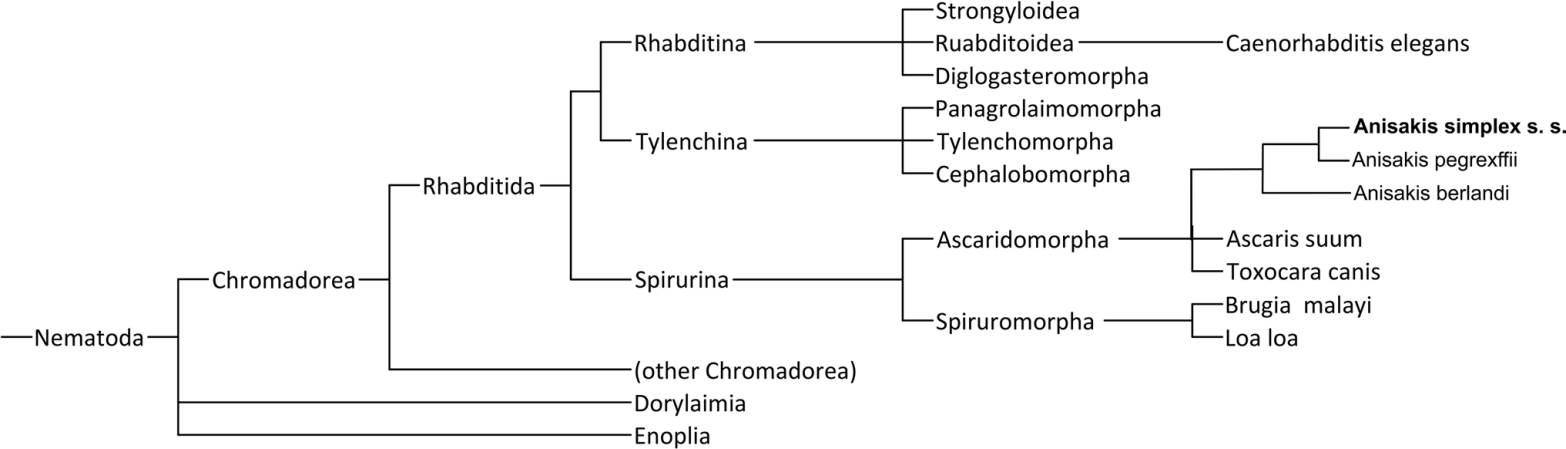
Phylogenetic representation of the evolutionary distances between different species of nematodes, based on the variability of the small ribosomal subunit 18S rRNA according to Mattiucci et al. [21]

This is the first study performed to select suitable reference genes for real-time PCR in *A. simplex* s. s. Previous research into the expression of target genes in *A. simplex* s. s. involved the normalization of results with the use of the reference gene chosen before for the model organism—free-living nematode, *C. elegans* [19]. The phylogenetic analysis of the 18S rRNA gene was carried out to show large phylogenetic distances, and thus little similarity, between *A. simplex* s. s. and other nematodes, especially *C. elegans*, based on which reference genes were chosen for *A. simplex* s. s. in most of the previously published studies. Therefore, the parasitic nematode *A. simplex* s. s. cannot be compared with free-living *C. elegans* and, thus, the endogenous controls for *A. simplex* s. s. were not used correctly, because the analyzed organism was more related to other parasitic nematodes than to free-living *C. elegans.* The presented phylogenetic analysis was aimed at highlighting these differences (Fig. 2) [20, 21]. Thus, we aimed to select for the first time reference genes for directly *A. simplex* sensu stricto, than for complex of three sister species (*A. simplex* s. s., *A. pegreffii* and *A. berlandi*), which are characterized by high transcriptomic, proteomic, geographic and pathogenic differences [22–24].

Our data are similar to the results of Strube et al. [25], where *ef-1α* was characterized by the lowest variation in expression in the study on *D. viviparus*. Trivedi and Arasu [26], who identified reference genes for *A. caninum,* and Li et al. [27], who analyzed *B. malayi,* reported that *actin* was the best endogenous control for the investigated organisms. Taki and Zhang [28] validated the stability of the reference genes for *C. elegans* and concluded that *ef-1α* was characterized by sufficiently stable expression to be used as a reference gene. In an analysis of gene expression in *C. elegans*, Hoogewijs et al. [29] identified *actin* and *gapdh* as genes with the least stable expression, which is consistent with the results noted for *A. simplex* (Fig. 1a, b). According to the literature data, at least two reference genes should be used for a given organism in every experiment [11]. Zhang et al*.* [30] observed that *ef-1α* could not be used as endogenous control for *C. elegans*. Taki and Zhang [28] arrived at the opposite conclusion, which indicates that endogenous controls should be determined in every experiment because of various factors (type of tissue, gene functions, method of isolation) affecting the expression of genes involved in the regulation of the basic cellular functions.

To validate the selection of reference genes in *A. simplex* s. s. under different experimental conditions, we checked the expression of *nhr-48* in two developmental stages following normalization with the proposed stable genes. Employing the most suitable gene (*ef-1α* and *ppi12*) to normalize *nhr-48* expression, we were able to demonstrate that the relative expression of the *nhr-48* gene was the same (0.17) in L3 stage and (0.37) in the L4 stage no matter which reference gene was used for normalization (Fig. 1c). The higher relative transcription level of *nhr-48* was noted in L4 developmental stage. *Nhr-48* expression results are a confirmation of the data obtained and combined from NormFinder and geNORM.

## Conclusions

In the current study, the translation elongation factor (*ef-1α)* and peptidyl-prolyl isomerase 12 (*ppi12)* were selected as the most stable reference genes for *A. simplex* s. s. This work will benefit future studies on gene expression in *A. simplex* s. s. and improve our understanding of the molecular characteristics of this parasitic nematode species.

## Electronic Supplementary Material

Below is the link to the electronic supplementary material.

Supplementary file1 Supplemental Table 1. Detailed data of the taxonomic identification based on highly specific amplification of the genomic DNA fragment ITS 1/2 (XLSX 25 kb)

## Data Availability

The data that support the findings of this study are openly available in NCBI at https://www.ncbi.nlm.nih.gov.
